# Combinations of Personal Responsibility: Differences on Pre-service and Practicing Teachers’ Efficacy, Engagement, Classroom Goal Structures and Wellbeing

**DOI:** 10.3389/fpsyg.2017.00906

**Published:** 2017-05-31

**Authors:** Lia M. Daniels, Amanda I. Radil, Lauren D. Goegan

**Affiliations:** Department of Educational Psychology, University of Alberta, EdmontonAB, Canada

**Keywords:** personal responsibility for teaching, teacher engagement, teaching efficacy, instructional practices, pre-service teachers, practicing teachers

## Abstract

Pre-service and practicing teachers feel responsible for a range of educational activities. Four domains of personal responsibility emerging in the literature are: student achievement, student motivation, relationships with students, and responsibility for ones own teaching. To date, most research has used variable-centered approaches to examining responsibilities even though the domains appear related. In two separate samples we used cluster analysis to explore how pre-service (*n* = 130) and practicing (*n* = 105) teachers combined personal responsibilities and their impact on three professional cognitions and their wellbeing. Both groups had low and high responsibility clusters but the third cluster differed: Pre-service teachers combined responsibilities for relationships and their own teaching in a cluster we refer to as teacher-based responsibility; whereas, practicing teachers combined achievement and motivation in a cluster we refer to as student-outcome focused responsibility. These combinations affected outcomes for pre-service but not practicing teachers. Pre-service teachers in the low responsibility cluster reported less engagement, less mastery approaches to instruction, and more performance goal structures than the other two clusters.

## Introduction

Pre-service and practicing teachers report feeling responsible for a host of activities including the developmental, social, and emotional needs of their students (Fischman et al., 2006; Lauermann, 2014). Although taking on these responsibilities is likely in the best interests of students, the impact of personal responsibility on teachers’ cognitions and wellbeing is unknown. The common negative picture of pre-service teachers struggling to make the transition to practice (Alberta Teaches’ Association, 2013) and practicing teachers as emotionally exhausted, overworked, stressed, and burned-out (Alberta Teachers’ Association, 1999; Martin et al., 2012), suggests that perhaps feeling highly responsible for students may come with some costs. This picture is not unique to Canada, with similar descriptions being recorded for teachers in many countries including the United States (National Commission on Teaching and America’s Future, 2007), England (Foster, 2017), and Australia (Australian Primary Principals’ Association, 2006). The purpose of this research was to look how different combinations of personal responsibilities relate to pre-service and practicing teachers’ sense of teacher efficacy, engagement, classroom goal structures, and wellbeing.

## Personal Responsibility

Lauermann and Karabenick’s (2011b) conceptualize personal responsibility as “as a sense of internal obligation and commitment to produce or prevent designated outcomes, or that these outcomes should have been produced or prevented” (p. 135). The Teacher Responsibility Scale (TRS) captures the multifaceted nature of responsibility in four domains “that teachers would consider highly relevant for their professional lives” (p. 17): personal responsibility for student achievement, student motivation, relationships with students, and responsibility for their own teaching. On the one hand, these four domains of personal responsibility are closely related to some of the domains of teaching reported as most stressful for teachers including high-stakes student testing, disruptive students, poor relationships, and being evaluated (Manthei et al., 1996; Kyriacou, 2001; Montgomery and Rupp, 2005). On the other hand, teachers also report high levels of satisfaction and other pleasant outcomes as extending from their responsibilities (Bishay, 1996; Lauermann, 2014). We were interested in how responsibilities relate to three cognitions (sense of efficacy, engagement, and classroom goal structures) as well as an indicator of wellbeing in separate samples of pre-service and practicing teachers.

### Outcomes Associated with Personal Responsibility

Sense of efficacy is defined as the extent to which a person feels capable to perform a task (Bandura, 2006). In the realm of teaching, sense of efficacy has been thoroughly researched with a high sense of efficacy often considered a marker of not only an effective teacher (Hattie, 2009; Klassen and Tze, 2014) but also a committed and less stressed teacher (e.g., Klassen and Chiu, 2011; Klassen and Durksen, 2014; Zee and Koomen, 2016). In creating the TRS, Lauermann and Karabenick (2013) demonstrated that although the four domains of personal responsibility were positively correlated with pre-service teachers’ sense of efficacy in the same four domains, they could not be collapsed into a single structural model thus suggesting they represent different constructs. They also showed that teachers had different mean levels of endorsement depending on the domain: they felt more efficacious for meeting students’ needs for motivation and achievement than they felt responsible for these domains; whereas, they felt more responsible than efficacious when it came to their own teaching.

It seems that personal responsibility may also impact teachers’ engagement. Generally, teachers who feel personally responsible tend to be internally motivated, self-regulated, proactive, concerned about others, and sensitive to the consequences of their own actions (Guskey, 1981; Pelletier et al., 2002; Fischman et al., 2006; Ryan and Weinstein, 2009; Lauermann and Karabenick, 2011b; Lauermann, 2014). In interviews, teachers distinguish responsible teachers from irresponsible ones based on their quality of engagement.

Responsible teachers do their “job every day not just when being observed” (#04HSq1). This included not only formal duties such as being on time, but also voluntary engagement: a responsible teacher “comes early, stays late and is never totally done with work” (#13HSq2). Irresponsible teachers, on the other hand, were characterized by work avoidance: they do “as little work as possible” (#03HSq4) and “they are late, demonstrate a lack of caring, make excuses and model inappropriate attitudes and actions. It is always someone else’s job or not important to take initiative, follow through on communication such as talks with parents, notes home, lesson plans, etc.” (#20ESq1B) (Lauermann, 2014, pp. 82–83).

These differences in quality of engagement may extend from the notion that personal responsibility is internalized and thus more adaptive than responsibility that is externally imposed. This distinction is important because teachers who experience high levels of being *held* responsible either in laboratory settings or through high stakes testing, administrative demands, and external mandates are more likely to rely on negative teaching strategies such as using more controlling language, providing less choice, and being more criticizing (Deci et al., 1982; Flink et al., 1990) than those who internalize responsibility in a personal way. Linking personal responsibility and classroom goal structures, Daniels et al. (2016) found that mastery-goal structures were negatively related to responsibility for achievement and positively related to responsibility for relationships; whereas, performance-goal structures were negatively related to responsibility for motivation. Lauermann (2013) found slightly different patterns whereby responsibility for motivation was positively related to performance goal practices, while responsibility for achievement and relationships predicted mastery goal practices. Berger et al. (2013) showed that of the four responsibilities for quality of teaching was positively related to autonomy supportive practices and structure and negatively related to classroom control and chaos.

Extending beyond their professional cognitions, teachers reported that although responsibility is a source of personal satisfaction and success for students it can also be a source of hard work and often requires personal compromise (Lauermann, 2014). Elsewhere, teachers have also described feelings of tension, stress, and even guilt arising from assuming too many responsibilities that they feel unable to meet (Broadfoot et al., 1988; Fischman et al., 2006). Although it seems that the positives associated with personal responsibility outnumber the negatives (Lauermann, 2014), there is also a possibility that a curvilinear relation exists between “felt responsibility and teachers’ wellbeing such that too much responsibility and identification with too many poorly defined roles may lead to such negative consequences as burnout and decreased job satisfaction” (Lauermann and Karabenick, 2011b, p. 133).

A limitation of the research reviewed above is that it relied exclusively on variable-centered analyses and thus failed to account for different combinations of personal responsibility. There is theoretical and empirical evidence that the four domains of personal responsibility are not completely separate and thus these sorts of combinations are important to consider.

### Theorized Relationships between Responsibilities

Although any combination of personal responsibility is possible, two theoretical perspectives may provide insight into particular combinations for teachers that may be more nuanced than simply low or high levels of all responsibilities. First, according to Weiner (1985) cognitions related to responsibility extend from attributions for controllability. Of the four domains of personal responsibility, it could be argued that teachers have more control over their own teaching and establishing relationships with their students than over students’ achievement and motivation. Thus, from an attributional perspective personal responsibilities for achievement and motivation may be more likely to be endorsed together because they represent outcomes that are more difficult for teachers to control. In turn, personal responsibilities for ones own teaching and relationships with students may be endorsed together because these are more proximal outcomes over which teachers can exert greater control.

The second framework that can be applied is Winter’s (1992) work conceptualizing responsibility as a dispositional construct consisting of two components. The first component is the “must” component characterized by an internal sense of obligation, adherence to a moral or legal standard, and self-judgment. The must component is proposed to focus on past outcomes and speculated to form first as a relatively immediate set of cognitions. The second component is referred to as the “social” or altruistic component characterized by concern for others and concern about the negative consequences of one’s own actions. This social component is concerned with future outcomes and their consequences for the self and others. Winter suggests that the social component may develop over time with greater cognitive awareness of possible consequences. Teachers are legally responsibility for the quality of their own teaching and for student achievement. Arguably, these could be the very first cognitions that pre-service teachers adopt when they choose to enter the profession and thus they may map onto Winter’s concept of “must” responsibility. In turn, personal responsibility for students’ motivation and relationships with students feel conceptually more focused on the future and concerns for students making them likely to map onto Winter’s “social” component.

The empirical data appears to favor the pairing based on Weiner (1985) more than Winter (1992). For example, when validating the scales Lauermann and Karabenick (2013) found that the correlation between responsibility for motivation and achievement amongst practicing teachers was *r* = 0.70 with the next highest relationship between responsibilities for relationships and own teaching at *r* = 0.53 (*r* = 0.78 and *r* = 0.70, respectively, for pre-service teachers). A sample of pre-service teachers (Daniels et al., 2016) also demonstrated that the relationship between responsibility for motivation and achievement was the strongest (*r* = 0.60) aligning with Weiner (1985) but also showed a strong correlation between achievement and own teaching (*r* = 0.55) aligning with Winter (1992). Working with vocational teachers Berger et al. (2013) showed a narrower range of correlations (*r*s = 0.22–0.48) with the association between responsibility for achievement and motivation suggested by Attribution Theory (*r* = 0.47) essentially equivalent to that of achievement and relationships (*r* = 0.48), a pairing that wouldn’t be predicted by either theoretical framework. These relationships suggest that there is value in taking a person-centered approach to examining personal responsibility. By doing so we can examine not just absolute quantity of responsibility but the combination of different responsibilities that maybe be particularly advantageous or not.

### Conceptual Framework for the Current Study

To date there is no quantitative evidence on how teachers’ combine personal responsibilities or on whether or not certain combinations of responsibility are more or less advantageous for pre-service and practicing teachers. We sought to bring empirical evidence to bear on whether or not personal responsibility is a double-edged sword by implementing a person-centered approach. The advantages of person-centered approaches have recently been highlighted (Wormington and Linnenbrink-Garcia, 2016) and many of those principles apply to the investigation of teachers’ personal responsibility. For example, variable centered-approaches to personal responsibility may be disadvantaged by the high correlation between responsibility for motivation and achievement. Because person-centered approaches examine responsibility at the level of the individual this correlation is less problematic. Also, once profiles of individuals are identified, researchers can make comparisons on outcomes of interest. We have elected to look for differences on teacher efficacy, teacher engagement, classroom goal structures, and a measure of wellbeing. For pre-service teachers we operationalized wellbeing as satisfaction with life in general and for practicing teachers we operationalized wellbeing as burnout.

## Materials and Methods

Both pre-service and practicing teachers participated in this research, which used a correlational self-report research design approved by the University’s Ethical Review Board. Consent was implied by completion of the survey. We had two research questions: (1) How do pre-service and practicing teachers’ combine their personal responsibilities? (2) Do these combinations result in different levels of sense of teaching efficacy, engagement, classroom goal structures, and wellbeing?

### Participants and Procedures

#### Pre-service Teachers

We recruited 139 pre-service teachers through a research participation pool. In exchange for 2.5% of their course grade participants completed a single online survey that contained questionnaires on personal responsibility as a teacher, sense of efficacy, engagement, classroom goal structures, and wellbeing. Participants ranged in age from 18 to 36 (*M* = 21.12 years) and were predominately females (82%). Eight participants did not indicate any racial or ethnic background information and the remaining self-reported to be of White European descent (80%) with a small group who identified themselves as Asian (10%). More participants were training to be secondary school teachers (53.8%) than elementary school teachers (41.5%) and eight did not indicate their level. Students represented a range of majors the largest groups being generalists (*n* = 38) followed by Biology majors (*n* = 22), English (*n* = 12), Physical Education (*n* = 11), and Mathematics (*n* = 10). We identified eight univariate outliers and one participant had no data, thus all analyses are based on a final sample of 130 pre-service teachers.

#### Practicing Teachers

Data were collected from a convenience sample of practicing teachers (*n* = 147) recruited during 2-days attendance at mandatory professional development conventions. At the request of research assistants who approached teachers with a clipboard, participants answered questionnaires related to their beliefs about personal responsibility as a teacher, sense of efficacy, engagement, and instructional practices. Participants were 22–72 years old (*M* = 38 years) and predominately females (82%). The majority of participants self-reported to be Canadian of European descent (82%), four participants indicated they were Aboriginal/First Nations, two Chinese, and 21 provided no information. Participants ranged in teaching experience from 0 to 40 years (*M* = 10.4 years). Of the total, 110 reported being full time teachers while the remaining indicated they were pre-service teachers attending the convention, part time, substitute teachers, or administrators. Six respondents were pre-school teachers, 74 taught elementary, 47 taught junior high school, 37 taught high school, suggesting that some teachers taught at more than one level. Participants also taught a wide range of subject areas including English Language Arts (21.8%), science (18.4%), math (17.0%), social studies (17.0%), and art (15%). In finalizing the sample we excluded five univariate outliers, one person with no data, and restricted the sample to those who were employed in a full time teaching position resulting in a sample of 104 teachers.

### Measures

#### Demographic Measures

Two demographic variables were included in our analyses for pre-service teachers: self-reported gender (*n* = 109 female, 20 male, 1 missing) and level of teaching (*n* = 54 elementary, 70 secondary, 6 missing). These two variables, plus years of teaching experience as a continuous variable were included for the practicing teachers: *n* = 90 female, 14 male; *n* = 49 elementary, 52 junior/senior high school, 3 missing; *M* = 10.12 years; *SD* = 8.42.

#### Responsibility

We used the TRS (Lauermann and Karabenick, 2013) to assess personal responsibility. For pre-service teachers the statement “Imagine that you have classes of your own” was added to the standard instructions “To what extent would you feel PERSONALLY responsible that you should have prevented each of the following?” Fourteen items measured the following four areas of personal responsibility: student motivation, student achievement, relationships with students, and their own teaching. Sample items and descriptive statistics for all variables are presented in **Table 1**.

**Table 1:** Descriptive statistics for all study variables.

Variable (number of items)	Sample item and response scale	Pre-service teachers *n* = 130				Practicing teachers *n* = 105			
			
		Range	*M*	*SD*	Alpha	Range	*M*	*SD*	Alpha
Responsibility for achievement (4)	I would feel personally responsible if a student of mine had very low achievement (1 = not at all; 7 = completely)	12–28	20.07	3.32	0.71	4–28	18.73	5.41	0.71
Responsibility for motivation (4)	I would feel personally responsible if a student of mine was not interested in the subject I teach (1 = not at all; 7 = completely)	5–26	15.74	4.53	0.83	5–28	17.24	6.06	0.93
Responsibility for relationships (3)	I would feel personally responsible if a student of mine thought he/she could not count on me when he/she needed help (1 = not at all; 7 = completely)	12–21	18.40	2.29	0.78	3–21	16.12	3.99	0.78
Responsibility for own teaching (3)	I would feel personally responsible if a lesson I taught was not as effective for student learning as I could have possibly made it (1 = not at all; 7 = completely)	14–21	19.02	1.73	0.62	9–21	17.14	2.96	0.86
Sense of teaching efficacy (11)	How confident are you that you [will be] are able to control disruptive behavior in the classroom? (1 = nothing; 9 = a great deal)	43–96	71.35	9.84	0.86	49–99	80.42	9.74	0.90
Teacher engagement (16)	While teaching, I [will] work with intensity. (1 = never, 4 = sometimes, 7 = always)	77–112	101.92	7.59	0.91	64–112	98.96	9.41	0.91
Classroom mastery structures (4)	Providing several different activities during class so that students can choose among them (1 = strongly disagree; 5 = strongly agree)	10–20	17.02	2.00	0.51	8–20	15.17	2.77	0.72
Classroom performance structures (5)	Giving special privileges to students who do the best work (1 = strongly disagree; 5 = strongly agree)	5–23	11.01	3.35	0.69	5–21	11.14	4.09	0.79
Measure of wellbeing^a^ (5 and 9)	Life satisfaction pre-service: In most ways, my life is close to my ideal (1 = strongly disagree; 7 = strongly agree) Burnout practicing: I feel frustrated by my job. (1 = never; 7 = always)	6–35	26.49	6.09	0.90	9–39	21.52	6.95	0.75


*^a^Wellbeing was operationalized as life satisfaction in pre-service teachers and burnout in practicing teachers.*

#### Teacher Sense of Efficacy

We used 11 of the 12 items from the short form of the Teacher Sense of Efficacy Scale (TSES), developed by Tschannen-Moran and Woolfolk Hoy (2001). We excluded the item “to what extent can you provide an alternative explanation or example when students are confused?” on the basis that it could not be easily adapted for pre-service teachers. In practicing teachers we excluded “How confident are you that you will be able to assist families in helping their children do well in school” because it has shown poor factor loading in other samples of Canadian teachers (Klassen et al., 2009). Although the TSES can be divided into efficacy for instructional strategies, classroom management, and student engagement, generally pre-service teachers lack sufficient experience to distinguish the factors and thus it is recommended to use a total sense of teaching self-efficacy score (Tschannen-Moran and Woolfolk Hoy, 2001). We created a single indicator of sense of teaching efficacy in both samples.

#### Engagement

We used the Engaged Teacher Scale (ETS; Klassen et al., 2013) to measure the degree of attention and absorption teachers feel (or pre-service teachers anticipate feeling) during teaching-related activities. In response to the directions “Below you will find a list of statements describing your experiences as a [future] teacher. Please indicate your personal response to each of these statements by selecting the number that best represents your answer,” participants rated items on cognitive engagement, emotional engagement, social engagement with students, and social engagement with colleagues. The ETS has been used as a single composite score for teacher engagement (Klassen et al., 2013; Durksen, 2015) as we have done here.

#### Instructional Practices

We used The Patterns of Adaptive Learning Scale (PALS; Midgley et al., 2000) to assess mastery and performance classroom goal structures. The items and instructions were adjusted slightly in order to accommodate pre-service teachers’ intentions: “The following items are about what type of classroom you intend to establish once teaching. Please think about things you plan to do when you have your own classroom”. Practicing teachers received the original instructions.

#### Indicators of Wellbeing

We operationalized wellbeing differently for pre-service and practicing teachers. Pre-service teachers responded to the five items on the Satisfaction with Life Scale (Diener et al., 1985). The scale is intended to measure general cognitive appraisals of one’s current satisfaction with life. Diener et al. (1985) offered cut-off scores to use as benchmarks of satisfaction: 31–35 extremely satisfied, 26–30 satisfied, 21–25 slightly satisfied, 20 neutral, 15–19 slightly dissatisfied, 10–14 dissatisfied, 5–9 extremely dissatisfied. The mean score reported by pre-service teachers in this sample was 26.49 (*SD* = 6.09) suggesting these participants were on average “satisfied.” For practicing teachers we operationalized wellbeing in terms of burnout and thus they rated nine items from an abbreviated version of the Maslach Burnout Inventory (McManus et al., 2002) tapping into emotional exhaustion, depersonalization, and personal accomplishment (1 = *strongly disagree* to 7 = *strongly agree*) rating scale. We created a composite score summing all nine items. Although this abbreviated version does not offer specific cut-off scores, the MBI Manual divides normative samples into thirds with each third corresponding to low, average, or high burnout (Maslach et al., 1996). Extrapolating from this practice, scores 9–27 would indicate low burnout, 28–54 would indicate average burnout, and 55–63 would suggest high levels of burnout.

### Rationale for Analyses and Hypotheses

All analyses were conducted separately for pre-service and practicing teachers. As preliminary analyses we correlated all variables to examine zero-order associations. Next we standardized the responsibility scales before conducting a *k*-means cluster analysis. We chose to use a *k*-means cluster analysis because we were interested in minimizing the variance within a cluster and maximizing variance between clusters (Huberty et al., 2005). *K*-means is a variance partitioning non-hierarchical clustering technique in which the number of cluster solutions is left to the discretion of the researcher and should be guided by theory and meaningful interpretation of the cluster solutions. We examined the resultant clusters from the theoretical perspectives of Weiner (1985) and Winter (1992) and expected either a three or four cluster solution to best describe the data with low and high responsibility clusters paired with one or two other theoretically grounded groupings. We examined each cluster in terms of adequately representing elementary and secondary level participants and females and males with chi-square tests. Finally, we used analysis of covariance to examine differences between the clusters on sense of efficacy, engagement, classroom goal structures, and wellbeing while covarying gender, teaching level, and, in the practicing teacher sample, years of teaching experience. We hypothesized the high responsibility cluster would have higher levels of sense of efficacy, engagement, mastery goal structures, and wellbeing but lower levels of performance instructional practices than the low responsibility cluster. We did not have *a priori* hypotheses for the other combinations of responsibilities. All significant main effects of cluster membership were followed up by testing all possible pairwise comparisons using *t*-tests and a conservative Bonferroni adjusted significance level (α = 0.05/5 = 0.01).

## Results

### Zero-Order Correlations

All correlations are presented in **Table 2**. The demographic variables were largely unrelated to the responsibility variables or the outcomes for pre-service teachers. The one exception was that female pre-service teachers were more inclined toward mastery approaches to instruction than males. For practicing teachers, gender and teaching level both correlated positively and significantly with responsibility for relationships such that women and secondary teachers felt more responsible in this domain. The longer participants had been teaching the more efficacious, engaged, and committed to mastery practices they reported feeling.

**Table 2:** Correlations for all study variables.

Variable	1	2	3	4	5	6	7	8	9	10	11	12
(1) Years teaching experience	—	0.03	0.07	-0.08	-0.14	-0.13	0.04	0.38**	0.26**	0.33**	-0.18	-0.39**
(2) Gender^a^	—	—	0.25**	0.15	0.11	0.26**	0.10	-0.10	0.06	0.13	-0.11	0.09
(3) Teaching level^b^	—	0.15	—	0.13	0.14	0.22**	0.11	-0.16	0.08	0.10	-0.14	-0.03
(4) Responsibility for motivation	—	0.01	-0.02	—	0.78**	0.47**	0.59**	-0.16	0.01	0.15	0.10	0.05
(5) Responsibility for achievement	—	-0.11	0.04	0.47**	—	0.54**	0.71**	-0.13	0.02	0.24**	0.02	0.06
(6) Responsibility for relationships	—	-0.01	-0.07	0.12	0.23**	—	0.65**	-0.05	0.13	0.27**	-0.15	-0.14
(7) Responsibility for own teaching	—	-0.04	-0.05	0.08	0.13	0.30**	—	-0.03	0.13	0.24**	-0.05	-0.07
(8) Teacher efficacy	—	0.13	-0.12	-0.14	-0.14	0.01	-0.11	—	0.57**	0.29**	0.10	-0.47**
(9) Teacher engagement	—	-0.17	-0.08	0.05	0.17	0.32**	0.30**	0.14	—	0.25**	0.03	-0.48**
(10) Mastery approaches	—	0.21*	-0.17	-0.12	-0.02	0.09	0.32**	-0.07	0.28**	—	0.01	-0.25*
(11) Performance approaches	—	0.08	0.08	0.07	-0.05	-0.05	-0.35**	0.05	-0.11	-0.16	—	0.13
(12) Measure of wellbeing^c^	—	0.26**	-0.04	-0.07	-0.01	0.04	0.01	0.22*	0.26**	0.04	-0.02	—


*Coefficients on the bottom half of the matrix are pre-service teachers and coefficients on the top half of the matrix are practicing teachers. ^∗^*p* < 0.05; ^∗∗^*p* < 0.01. ^a^(1 = male; 2 = female); ^b^teaching level (1 = elementary; 2 secondary); ^c^For pre-service wellbeing was measured by the Satisfaction with Life Scale (Diener et al., 1985). For practicing teachers wellbeing was measured by burnout (McManus et al., 2002).*

### Cluster Analysis

*K*-means cluster analyses were run separately specifying two-, three-, and four-cluster solutions. Based on the combinations supported by Attribution Theory (Weiner, 1985), we selected the three-cluster solution as the most meaningful for both pre-service and practicing teachers. Final cluster centroids representing the physical “center” of the cluster as defined by the average of all the scores constituting the cluster are presented in **Table 3**. For both samples, we labeled the first cluster *low responsibility* because it was characterized by centroids <0 for each domain of responsibility. The second cluster for both samples had the highest centroids and thus we labeled it *high responsibility*. The third clusters differed between pre-service and practicing teachers. For pre-service teachers we labeled the third cluster *teacher-focused responsibility* because it combined high levels of responsibility for relationships and own teaching with low responsibility for motivation and achievement. For practicing teachers, the third cluster had high levels of responsibility for motivation and achievement and a low level of responsibility for relationships and thus we labeled this cluster *student-outcome focused responsibility*.

**Table 3:** Cluster centroids, descriptive statistics, and pairwise comparisons on measures of personal responsibility.

Pre-service teachers										Pairwise comparisons		

Domain of responsibility	Cluster 1: low responsibility			Cluster 2: high responsibility			Cluster 3: high teacher focused responsibility			1 vs. 2	1 vs. 3	2 vs. 3
						
	*Centroid*	*M*	*SD*	*Centroid*	*M*	*SD*	*Centroid*	*M*	*SD*			
Achievement	-0.15	18.88	2.63	0.87	22.89	2.62	-0.38	18.12	2.45	-4.01^∗^	0.76	4.76^∗^
Motivation	-0.006	15.44	4.25	0.82	19.27	2.96	-0.74	12.17	3.18	-3.83^∗^	3.27^∗^	7.10^∗^
Relationships	-0.62	16.68	2.11	0.44	19.49	1.93	0.28	19.08	1.73	-2.81^∗^	-2.93^∗^	0.41
Own teaching	-0.72	17.34	1.32	0.46	19.75	1.22	0.61	20.05	1.73	-2.41^∗^	-2.71^∗^	-0.29
Sample information	*n* = 44; 15 elementary; 25 high school; 36 females, 8 males			*n* = 45; 19 elementary; 26 high school; 39 females, 6 males			*n* = 40; 20 elementary; 18 high school; 34 females, 6 males					

**Practicing teachers**										**Pairwise comparisons**		

**Domain of responsibility**	**Cluster 1: low responsibility**			**Cluster 2: high responsibility**			**Cluster 3: high student-outcome focused responsibility**			**1 vs. 2**	**1 vs. 3**	**2 vs. 3**
						
	***Centroid***	***M***	***SD***	***Centroid***	***M***	***SD***	***Centroid***	***M***	***SD***			

Achievement	-0.93	13.35	3.35	0.81	23.00	3.85	0.27	19.92	2.18	-9.65^∗^	-6.56^∗^	3.08^∗^
Motivation	-0.87	11.31	3.71	0.89	22.17	4.06	0.32	18.67	2.88	-10.87^∗^	-7.36^∗^	3.51^∗^
Relationships	-0.41	14.10	3.57	0.85	19.54	1.67	-0.53	13.58	3.35	-5.43^∗^	0.52	5.95^∗^
Own teaching	-0.55	14.92	2.67	0.80	19.46	1.53	-0.06	16.58	2.32	-4.54^∗^	-1.66^∗^	2.88^∗^
Sample information	*n* = 33; 16 elementary; 22 high school; 28 females, 5 males			*n* = 41; 21 elementary; 20 high school; 37 females, 4 males			*n* = 24; 11 elementary; 13 high school; 19 females, 5 males					


*^∗^*p* < 0.01.*

Neither the clusters of pre-service nor practicing teachers differed significantly from each other in terms of the distribution of teaching level or gender [pre-service teaching level, χ^2^(2) = 1.89, gender, χ^2^(2) = 0.41; practicing teaching level, χ^2^(2) = 1.38, *p* > 0.05, gender χ^2^(2) = 1.59, all *p*s > 0.05]. Because of our interest to also covary years of teaching experience in practicing teachers, we examined the distribution of experience across the three clusters. The low responsibility cluster was characterized by the most teaching experience, *M* = 12.29 years, *SD* = 9.15 years. The high responsibility cluster had *M* = 9.31 years, *SD* = 8.00 years and the student-outcome focused responsibility cluster had the fewest years, *M* = 8.35 years, *SD* = 7.78 years. These differences were not statistically significant: *F*(2,98) = 1.93, *p* > 0.05.

In each sample, we used four one-way ANOVAs to test for significant omnibus differences between the clusters (**Table 3**). For pre-service teachers all *F*-statistics were significant as were all but three follow-up pair-wise comparisons. The low responsibility and teacher-focused responsibility clusters did not differ on responsibility for achievement, *t*(124) = 0.76, *p* > 0.05, meaning these two clusters endorsed similar levels of this responsibility. The high responsibility cluster and the teacher-focused responsibility cluster did not differ on relationships *t*(124) = 0.41, *p* > 0.05 or own teaching *t*(124) = -0.29, *p* > 0.05, meaning that these two clusters represent similar levels of responsibility for relationships and own teaching but paired with either high or low levels of the other two domains of responsibility. For practicing teachers, all clusters differed from each other significantly except for the low responsibility cluster and the student-outcome focused responsibility cluster on responsibility for relationships. In other words, these two clusters reported similar levels of responsibility for relationships, *t*(101) = 0.52, *p* > 0.05. The means and standard deviations are presented as context for the pairwise comparisons (**Table 3**); however, they are not the primary source of interpretation for the clusters. Raw means can only be compared horizontally across clusters and not vertically within the cluster. Comparison of means across clusters, and cluster centroids within clusters, most accurately reflect the overall composition of the clusters and were used in determining the most suitable label for each cluster (Huberty et al., 2005).

### Cluster Profiles on Outcomes

For pre-service teachers three outcomes differed by cluster (**Table 4**). First, significant differences emerged for pre-service teachers’ engagement. Specifically, low responsibility pre-service teachers were significantly less engaged than high responsibility pre-service teachers (**Table 5**). The effect approached significance when comparing the low responsibility and teacher-focused responsibility clusters as well. A similar pattern appeared for mastery approaches to instruction whereby pre-service teachers in the low responsibility cluster reported significantly lower classroom mastery goal structures than pre-service teachers in either the high responsibility or teacher-focused responsibility clusters. Finally, the reverse pattern emerged for classroom performance structures. In this case pre-service teachers in the low responsibility cluster reported higher intentions establish classroom performance structures than pre-service teachers in the other two clusters. High responsibility did not differ from teacher-focused responsibility on any outcome. Likewise, none of the clusters differed on sense of teaching efficacy or satisfaction with life.

**Table 4:** Analysis of covariance.

Dependent variable	Cluster			Gender			Teaching level			Years experience		
						
Pre-service teachers	*M*s	*F*	η^2^	*M*s	*F*	η^2^	*M*s	*F*	η^2^			
Teacher efficacy	18.07	0.19	0.003	201.31	2.08	0.018	198.17	2.05	0.018	-	-	-
Teacher engagement	391.03	7.43**	0.11	118.05	2.24	0.019	12.73	0.24	0.002	-	-	-
Mastery	16.05	4.35**	0.07	13.02	3.53	0.029	6.28	1.70	0.014	-	-	-
Performance	64.33	6.28**	0.10	5.07	0.49	0.004	2.53	0.25	0.002	-	-	-
Satisfaction with life	30.97	0.86	0.01	325.24	9.01**	0.07	0.21	0.006	0.000			
Practicing teachers												
Teacher efficacy	80.07	1.12	0.03	45.48	0.64	0.01	301.38	4.23*	0.05	1207.85	16.95***	0.16
Teacher engagement	94.17	1.38	0.03	146.36	2.14	0.02	55.94	0.82	0.01	792.34	11.58**	0.12
Mastery	3.62	0.51	0.01	2.91	0.41	0.01	28.90	4.07*	0.04	95.23	13.41***	0.13
Performance	15.11	1.05	0.02	2.54	0.18	0.002	22.31	1.55	0.02	70.85	4.91*	0.05
Burnout	76.37	1.87	0.04	45.29	1.11	0.01	0.07	0.002	0.000	666.94	16.36***	0.16


*^∗^*p* < 0.05; ^∗∗^*p* < 0.01; ^∗∗∗^*p* < 0.001. The numerator *df* = 2 for cluster and *df* = 1 for gender, teaching level, and years experience in all *F*-tests. Covariates evaluated at gender = 1.15 and teaching level = 1.56 in pre-service sample at gender = 1.86, teaching level = 1.51 and years teaching experience = 10.15 for practicing sample.*

**Table 5:** Analysis of covariance: adjusted means and standard error for each cluster and significant pairwise comparisons in pre-service sample.

										Pairwise comparisons		

Outcome variable	Cluster 1: low responsibility			Cluster 2: high responsibility			Cluster 3: high teacher focused responsibility			1 vs. 2	1 vs. 3	2 vs. 3
						
	*n*	*M*	*SE*	*n*	*M*	*SE*	*n*	*M*	*SE*			
Teacher efficacy	37	71.60	1.63	44	70.43	1.48	38	71.55	1.60	—	—	—
Teacher engagement	40	98.74	1.15	43	104.91	1.11	37	101.96	1.2	-6.16^∗^	-3.22+	2.95
Mastery approaches to instruction	40	16.30	0.31	45	17.19	0.29	38	17.54	0.31	-0.89^∗^	-1.25^∗^	0.35
Performance approaches to instruction	40	12.68	0.51	45	10.61	0.48	38	10.35	0.52	2.07^∗^	2.33^∗^	0.25
Satisfaction with life	40	25.50	0.95	44	27.14	0.91	38	26.84	0.98	—	—	—


*^∗^*p* < 0.01 and + = 0.057. Covariates are evaluated at gender = 1.15 and teaching level = 1.56.*

For practicing teachers, contrary to our assumptions, no significant main effects emerged for cluster on any outcome variable (**Table 4**). Level of teaching had a significant effect on sense of teaching efficacy such that high school teachers reported higher feelings of efficacy (*M* = 82.98, *SD* = 7.79) than elementary school teachers (*M* = 78.34, *SD* = 10.10). Level of teaching also had a statistically significant effect on classroom mastery structures such that high school teachers reported lower endorsement of mastery structures (*M* = 14.63, *SD* = 2.81) than elementary school teachers (*M* = 15.64, *SD* = 2.72). As suggested by the correlations, there was also a significant relationship between years teaching experience and each outcome such that years of experience was associated with more efficacy, engagement, mastery goal structures, and fewer performance goal structures. Teachers with more experience also reported higher levels of burnout.

## Discussion

The purpose of this research was to examine how pre-service and practicing teachers combine their personal responsibilities and evaluate if particular combinations are more advantageous for specific outcomes. Different stories emerged for pre-service compared to practicing teachers both in terms of how personal responsibilities were combined and how personal responsibility related to outcomes. We focus on how the results are similar or different for pre-service and practicing teachers and what this implies for the developmental trajectory of teachers. First we discuss why a *teacher-focused responsibility* cluster emerged for pre-service teachers whereas a *student-outcome focused responsibility* cluster emerged for practicing teachers. Second, we examine the impact of different clusters on outcomes. Third, we explore the relative levels of endorsement in each domain of responsibility. Finally, we examine the limitations of this research and propose directions for future research.

### Differences in Clusters between Pre-service and Practicing Teachers

The cluster solutions differed for pre-service and practicing teachers. A group of pre-service teachers emerged who reported higher levels of responsibility for their own teaching and their relationships with students than for students’ motivation and achievement. From the perspective of Attribution Theory (Weiner, 1985), this cluster may be distinguishing responsibilities for which they can exert personal control, namely how well they teach and what kinds of relationships they establish with students, from responsibilities that they are less able to control, namely how motivated students are and their levels of achievement. In contrast, for practicing teachers an opposite group emerged that emphasized student motivation and achievement over responsibilities for teaching and relationships. One explanation for this difference may be the context in which pre-service versus practicing teachers exist: On a daily basis pre-service teachers are students, learning to teach, and presumably looking forward toward their career. In contrast, on a daily basis practicing teachers face the demands of students, parents, and other stakeholders with the opportunity to focus on past events. These contexts may influence the responsibilities that are most salient for each group. Pre-service teachers may focus on why they chose to pursue teaching, many of whom did so to help children (Beck and Kosnik, 2002) perhaps heightening their sense of personal responsibility to maintain relationships (Lauermann and Karabenick, 2011a). Similarly, as students, pre-service teachers are learning to be “good” teachers making the responsibility for their own teaching one that they consider regularly. In contrast, practicing teachers may experience more frequent reminders that they are responsible for student achievement and as they face classroom management challenges (Evertson and Weinstein, 2006) they may understand how important student motivation is in sustaining relationships and supporting achievement. Of course our samples of pre-service and practicing teachers are separate cohorts and do not represent a longitudinal sample. As such, although we can record these differences, they do not represent a truly developmental trajectory.

### Impact of Cluster Membership on Outcomes

For pre-service teachers, although membership in either the high responsibility cluster or the teacher-focused responsibility cluster implied different priorities from a responsibility perspective, they appear to have the same consequences in terms of pre-service teachers’ outcomes. Either high responsibility in all domains or feeling dominantly responsible for ones own teaching and relationships resulted in greater levels of engagement, more support of classroom mastery goal structures, and less support of classroom performance goal structures than being in the low responsibility cluster. In other words, our evidence suggests that more responsibility is more beneficial for pre-service teachers than less responsibility. Two possible explanations come to mind. First, from the perspective of teacher identity, pre-service teachers who have internalized more domains of personal responsibility may be showing a more fully developed professional identity than those who report lower levels of these responsibilities (Sutherland et al., 2010). It has been argued that “what may result from a teacher’s realization of his or her identity, in performance within teaching contexts, is a sense of agency, of empowerment to move ideas forward, to reach goals or even to transform the context” (Beauchamp and Thomas, 2009, p. 183). This association between identity and agency can explain why pre-service teachers in the high or high-control responsibility clusters felt more engaged and mastery focused and less performance focused than those in the low cluster. Second, it is possible that pre-service teachers are unrealistically optimistic about their abilities to meet these responsibilities (Weinstein, 1989; Biddle, 1997). In other words, because they are not yet in full-time teaching positions pre-service teachers do not bear the full weight of the personal responsibilities or have to deal with failing to meet them. Indeed Lauermann and Karabenick (2011b) postulated that a curvilinear relationship may emerge when assumed responsibilities cannot be met – a reality that these pre-service teachers have not yet had to face.

In contrast, for practicing teachers, there were no statistically significant differences between clusters on any outcome. Instead, it seems that for this sample years of teaching experience outdid any combination of responsibility. Teachers with more experience reported higher levels of efficacy, engagement, classroom mastery goal structures along with lower levels of classroom performance goal structures and more burnout. On the one hand this paints a picture of experienced teachers who feel competent in their professional roles and choose adaptive instructional practices for their students and yet still suffer from symptoms of burnout. On the other hand, this suggests that novice teachers are less confident and appear less inclined to use adaptive instructional practices (Pillen et al., 2013; Daniels, 2015). If they are able to persist, experience in and of itself can help alleviate these negative feelings for novice teachers, but the ability to persist is more tenuous and our results do not help us better understand how to support novice teachers from the perspective of balancing their personal responsibilities.

### Mean Level Endorsement of Personal Responsibilities

Despite the emergence of different combinations of responsibilities, the rank order endorsement of each responsibility was the same for pre-service and practicing teachers: Both samples most strongly reported feeling personally responsible for their own teaching, followed by relationships, then achievement, and finally for student motivation. This order of endorsement has been recorded in previous research (Berger et al., 2013; Lauermann, 2013; Daniels et al., 2016) and raises concerns about why teachers report such low levels of responsibility for student motivation particularly in an educational climate where intrinsic motivation is often in short supply (Legault et al., 2006; Usher and Kober, 2012). One possible explanation is that pre-service and practicing teachers may view motivation as an innate quality that cannot be influenced (Dweck, 2006). This is an empirical question that could be answered through future research and would provide valuable insight into understanding why certain responsibilities appear to be routinely endorsed less than others.

It is also interesting to note that pre-service teachers had a wider range of scores with nearly 2.5 points on the seven point scale separating their highest and lowest domains of personal responsibility. In contrast, practicing teachers had a much narrower range with about 1.5 points separating their lowest and high domains. Extant research shows that pre-service teachers may overestimate their capacities in comparison to practicing teachers (e.g., Pendergast et al., 2011) in part because they do not fully understand the realities of the classroom (Lortie, 1975). Longitudinal research would be helpful to figure out if pre-service teachers temper their endorsement of responsibilities when they begin teaching as they have done for other beliefs (e.g., Daniels, 2015).

### Limitations and Directions for Future Research

The results of this research need to be interpreted in light of the following four limitations. First, both studies relied exclusively on self-report data. Although this is an efficient way to bring data to bear on the current research questions, future research may want to augment this perspective with observations of teaching practices, student evaluations of teacher engagement, and administrator perspectives. Second, we chose our clusters for theoretical and not statistical reasons. Thus, these clusters need to be replicated in independent samples and using more contemporary approaches to examining profiles such as latent class analysis which was precluded by our limited sample size. Third, the research presented herein represented a snapshot of responsibility in separate samples of pre-service and practicing teachers and not longitudinal data. Future research could benefit from longitudinal designs that advance our understanding of when pre-service teachers begin to adopt these responsibilities, if they shift in terms of how they are combined and when that occurs, and whether or not mean level endorsement changes over time. This would also allow investigations into possible curvilinear effects of responsibility as suggested by Lauermann and Karabenick (2011b). Fourth, although we selected outcomes that are often associated with effective teaching (e.g., Hattie, 2009) they do not allow us to infer how teachers’ personal responsibilities impact students’ actual outcomes. Future research would be wise to collect student achievement data and connect this to teachers’ personal responsibilities.

## Conclusion

The implications of the current results suggest that teacher education programs should help pre-service teachers to assume personal responsibilities associated with the profession because during their training more responsibility is associated with more effective cognitions. Once practicing, it seems that time will contribute more meaningfully to their professional and personal outcomes than focusing on specific responsibilities either in isolation or in combination. Both of these perspectives provide ample room for future research and particularly highlight the importance of examining personal responsibility in both pre-service and practicing teachers because important differences may exist in terms of how personal responsibility is developed.

## Ethics Statement

This study was carried out in accordance with the recommendations of Research Ethics and Management Online (REMO) System at the University of Alberta. The specific protocol approval numbers are: Pro00054513 and Pro00035849. An Information Letter explaining participants’ rights was presented at the beginning of the questionnaire and then informed consent was given when participants began the questionnaire.

## Author Contributions

LD was the lead author in conceptualizing the research and writing the manuscript. AR and LG contributed to all steps of the research and then critically reviewed and revised the manuscript. All authors accept accountability for the final version of the manuscript.

## Conflict of Interest Statement

The authors declare that the research was conducted in the absence of any commercial or financial relationships that could be construed as a potential conflict of interest. The reviewer LIFF and handling Editor declared their shared affiliation, and the handling Editor states that the process nevertheless met the standards of a fair and objective review.
